# Comparative Evaluation of Human Mesenchymal Stem Cells of Fetal (Wharton's Jelly) and Adult (Adipose Tissue) Origin during Prolonged *In Vitro* Expansion: Considerations for Cytotherapy

**DOI:** 10.1155/2013/246134

**Published:** 2013-03-03

**Authors:** I. Christodoulou, F. N. Kolisis, D. Papaevangeliou, V. Zoumpourlis

**Affiliations:** ^1^Institute of Biology, Medicinal Chemistry & Biotechnology, National Hellenic Research Foundation (NHRF), 48 Vasileos Konstantinou Avenue, 11635 Athens, Greece; ^2^School of Chemical Engineering, National Technical University of Athens, 15780 Athens, Greece; ^3^TAK-EIE Stem Cell Bank S.A., NHRF, 11635 Athens, Greece

## Abstract

Mesenchymal stem cells (MSCs) are somatic cells with a dual capacity for self-renewal and differentiation, and diverse therapeutic applicability, both experimentally and in the clinic. These cells can be isolated from various human tissues that may differ anatomically or developmentally with relative ease. Heterogeneity due to biological origin or *in vitro* manipulation is, nevertheless, considerable and may equate to differences in qualitative and quantitative characteristics which can prove crucial for successful therapeutic use. With this in mind, in the present study we have evaluated the proliferation kinetics and phenotypic characteristics of MSCs derived from two abundant sources, that is, fetal umbilical cord matrix (Wharton's jelly) and adult adipose tissue (termed WJSC and ADSC, resp.) during prolonged *in vitro* expansion, a process necessary for obtaining cell numbers sufficient for clinical application. Our results show that WJSC are derived with relatively high efficiency and bear a substantially increased proliferation capacity whilst largely sustaining the expression of typical immunophenotypic markers, whereas ADSC exhibit a reduced proliferation potential showing typical signs of senescence at an early stage. By combining kinetic with phenotypic data we identify culture thresholds up to which both cell types maintain their stem properties, and we discuss the practical implications of their differences.

## 1. Introduction

Mesenchymal stem cells (MSCs) are somatic cells with an ability to self-renew and to differentiate towards a variety of specialized cell types through a combination of symmetric and asymmetric divisions. Populations of MSCs can be relatively easily isolated from a number of tissues that may differ both developmentally (e.g., fetal versus adult) and anatomically (e.g., bone marrow versus fat). Due to their unique properties of self-renewal and multipotency, as well as their immunogenic profile [1–3] (in many cases they have been shown to be hypoimmunogenic and/or can be transplanted autologously following *ex vivo* expansion), they have been utilized in several therapeutic applications such as tissue repair and regeneration and autoimmune disease over the last decade [4]. 

Despite the broad commonalities in defining characteristics and clinical applicability potential, there are unquestionable qualitative and quantitative differences in terms of their isolation efficiency and *in vitro* manipulation performance, as well as their efficacy in animal models and clinical studies, which have been well highlighted in a number of publications [5–8]. Indeed, the process of getting MSCs from the tissue source to the patient is far from streamlined and includes several factors of heterogeneity, the main ones relating to isolation and expansion of these cell populations [9]. In particular, lack of consistency/consensus in cell manipulation (i.e., diversity in the isolation and culture protocols applied), in addition to inherent heterogeneity in the samples (related to donor tissue origin, age, sex, and underlying pathology), may have an impact on the quality and quantity of isolated cells. Moreover, MSCs (in contrast to embryonic stem cells) are bound to have, to a lesser or greater extent, a limited replication potential in an artificial *in vitro* culture environment. Indeed, Hayflick's theory states that most somatic cell types may undergo a maximum of 70 population doublings before reaching replicative senescence [10, 11], and MSCs are bound to be constrained by this limitation. Nevertheless, in the case of MSCs-based cytotherapy, it is estimated that approximately 1–5 million cells/kg body are required for successful outcome; therefore a degree of *ex vivo* expansion is necessary, and this holds true in over 60% of cases of cellular transplants [12, 13]. A question therefore arises whether MSCs can sustain their expansion potential and phenotypic stability over prolonged culture periods, for how long, and whether possible disparities exist between diverse populations, rendering some of them more competent and suitable for consideration in cytotherapy protocols. 

In the present study we have focused on the characterization of postnatal human MSC populations originating from two sources that differ both developmentally, as well as anatomically, that is, cells isolated from the matrix (Wharton's jelly) of the fetal umbilical cord (UC) tissue (termed WJSC) [14] and from the abdominal adipose tissue of adults (adipose-derived; ADSC) [15]. The main advantages of these MSC sources compared to other stem cell origins are (a) abundant cell availability, (b) relative ease of access for stem cell isolation with minimal or no tissue morbidity, and (c) an immunogenic profile of isolated cells that is favorable for cellular transplantation [16, 17]. 

The main hypothesis behind this work has been that there are differences in qualitative and quantitative characteristics of these MSC populations, in terms of their adaptation to extended culture, which ultimately relate to practical considerations regarding their clinical use. This study which, to our knowledge, is the first to address this issue describes a culture regime that sustains the MSC phenotype, while highlighting a threshold for optimal cell expansion.

## 2. Materials and Methods

### 2.1. MSC Isolation 

#### 2.1.1. WJSC

WJSC were isolated from donated UC tissue samples from normal, full-term deliveries following approval by the institutional bioethics review board. Umbilical cords were transported to the lab in Hanks Balanced Salt Solution (HBSS) containing 1% penicillin/streptomycin solution (both from Invitrogen), and cell isolation was carried out within a maximum of 48 hrs from tissue collection according to a modified protocol based on that described by Seshareddy and colleagues [18], using reagents free of animal products. Briefly, the blood vessels were carefully removed, and the UC was minced into 1-2 mm^3^ fragments and then incubated with a mixture of 0.26% collagenase type I (Biochrom) and 0.07% hyaluronidase (Applichem) for 2 hrs, followed by 0.125% trypsin (Biochrom) for 30 min with gentle agitation at 37°C. The digested mixture was then passed through a 100 *μ*m cell strainer (BD) and washed in PBS (Biochrom) to obtain cell suspensions that were stained with 0.4% trypan blue (Biochrom), counted on a hemocytometer, aliquoted, and frozen down in liquid N_2._

#### 2.1.2. ADSC

ADSC were isolated from liposuction aspirates donated for research purposes which were obtained from subcutaneous adipose tissue sites of subjects (age: 44 ± 11 yrs; sex: 71/29% male/female) undergoing elective procedures in local plastic surgery offices. The research protocol used had been approved by the institutional bioethics review board. Cell isolation was carried out under xeno-free conditions within 48 hrs from collection following a modified protocol based on that described by Zuk et al. [19]. Briefly, lipoaspirate samples were first washed in the collection bag using 250 mL normal saline, and after removal of the blood fraction, adipose tissue was dispersed by digestion with 0.2% collagenase type I (Biochrom) at 37°C for 1 hr with agitation. After one more wash, the layer below the floating lipid-filled adipocytes was dispensed into 50 mL tubes and centrifuged at 900 g for 45 min. Subsequently, the supernatant was aspirated and the cell pellets (stromal vascular fraction (SVF)) frozen down in liquid N_2_, after determining cell numbers with a hematology blood analyzer (Beckman-Coulter). 

### 2.2. MSC Culture 

#### 2.2.1. WJSC

The five best samples (i.e., with the highest number of nucleated cells) out of a pool of 34 samples were resuscitated, plated on noncoated 75-cm^2^ flasks (Corning), and cultured in growth medium (GM), which consisted of DMEM/F12 (with 3.5 g/L glucose, ultraglutamine I, and Na pyruvate; Lonza) supplemented with 10% batch-tested fetal bovine serum (FBS), 15 mM HEPES, 1x nonessential amino acids, and 1% penicillin/streptomycin (all from Invitrogen). Preliminary experiments testing DMEM and DMEM/F12-based media (both used in the literature for maintenance of WJSC, as well as ADSC cultures) had established DMEM/F12 containing stable L-glutamine dipeptide as the optimal basal medium for propagation of both MSC types (see Section 3). Three days after initial plating, nonadherent cells were removed and the medium was replaced with fresh GM. Cells were maintained in a humidified atmosphere with 5% CO_2_ in air at 37°C with media changes (complete) every 3-4 days until 70%–80% confluence. Subcultivation (passaging) was carried out by trypsinization using 0.05% trypsin-EDTA solution (TE; Invitrogen) and replating the cells at a density of 4,000 cells/cm^2^ in 75 cm^2^ flasks. Frozen stocks of 0.5–2 million cells were maintained in 10% DMSO in FBS in 2 mL cryovials (Nunc) stored in liquid N_2_.

#### 2.2.2. ADSC

The eight best samples (i.e., with the highest number of nucleated cells) out of a pool of 29 samples were resuscitated, plated on noncoated 25 cm^2^ flasks (Greiner) and cultured in GM, as described previously. After three days of culture, nonadherent cells were removed and the medium was replaced. Adherent cells were grown at 37°C in 5% CO_2_ at saturating humidity, with medium changes twice weekly. Once 70%–80% confluence was reached, cells were detached with 0.05% trypsin-EDTA and replated (passaged) in 75 cm^2^ flasks at a seeding density of 6,000 cells/cm^2^. Frozen stocks of 0.5–2 million cells/cryovial were maintained in liquid N_2_, as described previously.

### 2.3. Determination of Proliferation Kinetics and Cell Size

The number of doublings in cell populations (PD) and the time period (in hrs) in which they occur (PDT) were calculated according to the following formulas:

(1) $$\begin{array}{c} \text{PD}\;=\;\left(\frac{1}{\log_{10}\;2}\right)\times \log_{10}\left(\frac{\text{Nt}}{\text{No}}\right),\\ \text{PDT}\;=\;t\;\;\times \;\left[\frac{\log_{10}\;2}{\left(\log_{10}\;\text{Nt}-\log_{10}\;\text{No}\right)}\right], \end{array}$$

where No is number of viable (as determined by trypan blue exclusion) cells at seeding, Nt is number of viable cells at harvest, and *t* is time (in hrs) between seeding and harvest. A proliferation index (PI) was used as a standardized measure of the proliferative capacity of the cells. PI was defined as the ratio of the number of population doublings (PD) in a specific passage over the respective time period (PDT) in which the former take place. 

To determine cell size (surface area, in *μ*m^2^), photos of six random, nonoverlapping fields were captured at 10x magnification using an Olympus E520 digital camera mounted on an Axiovert 40 CFL (Zeiss) inverted microscope. The area of at least five different cells per field was measured using Adobe Photoshop CS5 Extended. Cells with sizes in the top 25% of each size range were considered as “giant” cells (>4600 *μ*m^2^ and >3880 *μ*m^2^, for ADSC and WJSC, resp.). 

### 2.4. Characterization of Isolated Stem Cell Populations

#### 2.4.1. Immunophenotyping

Cultured WJSC and ADSC were characterized for the expression of the following markers: CD29 (*β*_1_-integrin), CD44 (H-CAM), CD73 (ECTO-5′ nuclease/SH3), CD90 (THY-1), CD105 (endoglin/SH2), CD14 (LeuM3/MY4), CD34 (HPCA1/gp105–120), and CD45 (LCA). Cells were detached using StemPro Accutase (Invitrogen), washed in PBS and stained using specific FITC-, PE-, ECD-, or PC5-conjugated monoclonal antibodies or isotype-matched (IgG1a/IgG2a) controls (all by Beckman-Coulter, except CD105-PE which was purchased from BD). 7-AAD (Beckman-Coulter) was used as a viability stain. Analysis was performed on a Beckman-Coulter EPICS XL-MCL flow cytometer. At least 20,000 live events were acquired.

#### 2.4.2. CFU-F Assay

The capacity of ADSC and WJSC for self-renewal was evaluated by seeding cells at clonal densities (20–80 cells/cm^2^) in 10 cm dishes and fixing and staining the resulting CFU-F two weeks later with 0.5% crystal violet/methanol solution (Digirolamo et al., 1999) [20]. Colonies > 2 mm in diameter were counted manually.

#### 2.4.3. ALP Assay

Alkaline-phosphatase-(ALP-)-positive CFU, which represent clonal populations of cells with osteoprtogenitor properties, were detected in duplicate cultures of ADSC/WJSC (set up as described for CFU-F assay previously mentioned) using the Sigma leukocyte ALP histochemistry staining kit (Kaplow, 1968) [21], following the manufacturer's recommendations. Colonies counterstained with hematoxylin and those > 2 mm in diameter were counted manually.

#### 2.4.4. Osteogenic Differentiation

 The potency of WJSC and ADSC for directed differentiation towards osteoblast lineage was tested by alizarin red staining of mineralized bone nodules [22]. Cells were grown in triplicate in 6-well plate for 21 days in the presence of osteogenic supplements (50 *μ*g/mL *β*-glycerophosphate, 10 mM L-ascorbic acid 2-phosphate, and 5 × 10^−7^ M dexamethasone; all from Sigma) and then fixed in 10% formol saline and stained with 2% alizarin red. Bone nodules > 1 mm were manually counted and scored in three different categories: + (< 20), ++ (20–50), and +++ (> 50).

#### 2.4.5. Calcium Assay

The Ca content of mineralized culture lysates was measured by a colorimetric assay (Cayman) [23], following the manufacturer's recommendations. Cells were seeded at a density of 500/cm^2^ in duplicate in 10 cm dishes and grown in the presence of osteogenic supplements (see aforementioned) for 21 days. Following a wash in Ca/Mg-free PBS they were lysed in 100 mM ice-cold Tris buffer, spun at 10,000 g, and the supernatants stored at −80°C until used. Sample absorbances were measured at 570 nm using a Tecan microplate reader and compared across a reference curve constructed using calcium standards. The assay detection limit was 0.5 mg/dL.

#### 2.4.6. Senescence Assay

Cellular senescence was determined by *β*-gal staining of subconfluent day 4-5 cultures set up in 6-well plates, using the Sigma cell senescence histochemical kit [24], according to the manufacturer's instructions. Briefly, cells were fixed and incubated with the staining mix containing X-gal overnight at 37°C in a low CO_2_ environment. The occurrence of *β*-gal positive blue-green cells was determined by counting a minimum of 50 cells in at least five random, nonoverlapping fields under a microscope. Each assay was performed in duplicate.

#### 2.4.7. Adherence Assay

Cell detachment was measured in time series experiments set up in 6-well plates in triplicate. Cells were seeded at 4,000–6,000/cm^2^ and left to grow to 70%–80% confluence, upon which they were washed in PBS and incubated with 600 *μ*L warm 0.05% trypsin-EDTA (TE) per well for up to 30 min without agitation. Every 5 min, the digested cell suspensions were drawn out into 1.5 mL tubes, and an aliquot was mixed with trypan blue and live cells counted on a Neubauer plate, while 600 *μ*L of fresh, warm TE was added per well for the next measurement. Control cells were incubated with TE at 37°C with frequent tapping until 100% detached from culture plastic. 

### 2.5. Statistical Analysis

Parametric and nonparametric tests (Spearman correlation analysis, Mann-Whitney pairwise data comparisons, and ANOVA) were conducted using GraphPad Prism 5.04 statistical analysis software. Data are depicted as means ± SD, unless otherwise stated. A *P* value < 0.05 was considered as statistically significant.

## 3. Results

### 3.1. Primary Cell Isolation and Culture Initiation Efficiency

#### 3.1.1. WJSC

**Figure 1:**
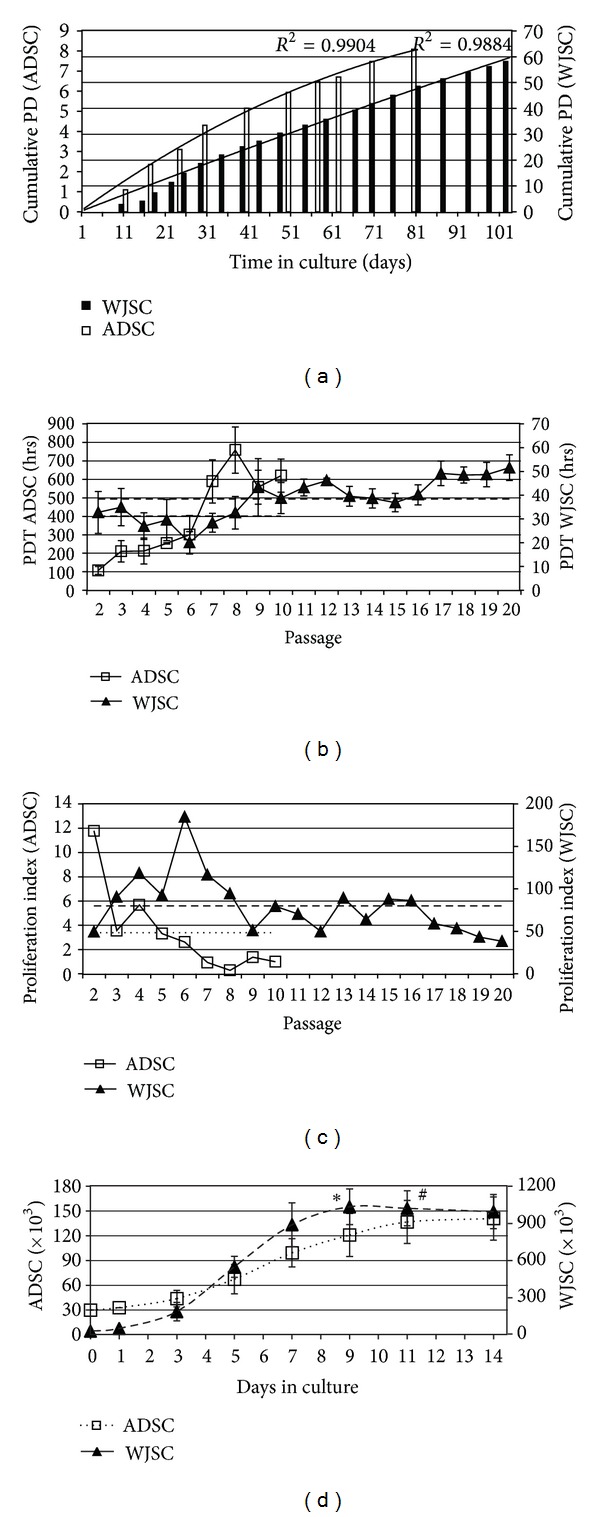
Proliferation kinetics of WJSC and ADSC over extended *in vitro* propagation. (a) Number of cumulative population doublings (PD) as a function of time in culture; (b) cell population doubling times—PDT—(hrs) at respective passages; (c) proliferation index (PI; ratio of PD over respective PDT); (d) growth curve of WJSC and ADSC within a single passage (3 to 4). The ∗ and # symbols highlight the points when confluence was reached for WJSC and ADSC, respectively. Dashed and long dashed horizontal lines denote mean values for ADSC and WJSC, respectively.

Mean nucleated cell yield following UC tissue processing was about 2.3 × 10^6^ and was found to positively correlate (Spearman *r* = 0.722, *P* < 0.001) with tissue properties (i.e., UC length) (Table 1 and see in Supplementary Material available online at http://dx.doi.org/10.1155/2013/246134 Figure 1). The isolation protocol involved removal of the vessels that due to the supercoiled anatomy of the UC increases tissue processing time and hence results in some cell loss compared to protocols based on digestion of whole UC. Nevertheless our protocol produces a more homogeneous population than the latter which results in the isolation of a mixed population of at least four stem cell types [25, 26]. Five out of five (100%) cultures of adherent cells derived from plated UC nucleated cell samples led to the establishment of cultures that grew successfully beyond passage 3 (Table 1). The three of these cultures with the shortest PDT (see the following) were further subcultured and used for the experiments described in this study. The percentage of adherent cells in the primary cultures was estimated at 12%.

#### 3.1.2. ADSC

For ADSC, mean nucleated cell yield at isolation was 312 × 10^6^ cells (Table 1). Regression analysis of samples showed that this yield was not significantly associated with initial tissue quantity (donor adipose weight) (see Supplementary Figure 1) or other donor characteristics, such as age or sex. Nevertheless there was a strong linear correlation of the number of isolated cells with adipose tissue weight for samples up to 200 g (Spearman *r* = 0.952, *P* < 0.001), highlighting the increased efficiency of the protocol for samples in that range. The eight adipose tissue samples with the highest mononuclear cell yields were plated out and used to establish cultures of ADSC. Five of these samples tested proliferated very slowly (less than 20% increase in cell yield between passages) and failed to grow beyond passage 3, whereas 3/8 (37.5%) proliferated further and were used for the experiments. ADSC yield was approximately 1.8 × 10^6^ cells/g lipoaspirate, with the percentage of adherent cells in the primary cultures being much lower than that of WJSC at 0.93%. This yield is slightly higher than that reported elsewhere (1 × 10^6^ cells/g) [27]. Viability of isolated cells and resuscitated cells following initial cryopreservation was consistently high (>92%) and similar to that of WJSC (Table 1). 

### 3.2. Proliferation Kinetics of Long-Term Cultures

Figures 1(a)–1(c) depict the proliferation dynamics of the two MSC populations during prolonged *in vitro* subcultivation, while Table 2 summarises the main findings. The mean cell yield shown in Table 2 is the one that arises from the serial propagation of cells following the subculture protocol described in Section 2. This was higher (by more than twofold) for WJSC and related to a constant number of about three PD per passage (depicted by the linearity of the graph in Figure 1(a)) accumulating to a total of 58 PD in the 100-day culture period. Mean PDT was 38.4 ± 8.6 hrs over that period. Peak proliferative rate was observed at passage 6 (considering PI and PDT; Figures 1(b) and 1(c), resp.), which equated to nearly 19 PD over a month in culture. Maximum PI of WJSC (observed at passage 6) was over 15-fold higher than that of ADSC and coincided with a maximum number of 3.72 ± 0.32 PD. WJSC showed no signs of slowdown even after 15 passages (45 PD) (*P* > 0.05, ANOVA on PDT of passages 2–16). There was nevertheless a significant increase in PDT after passage 17, which exceeded 50 hrs by passage 20. 

 ADSC proliferation capacity was maximum at passage 2 (see Figures 1(b) and 1(c)) and remained above average until passage 5, although related to a poor number of cumulative PD (just over five). Signs of senescence and essentially cessation of proliferation appeared after passage 6. Thenceforth a plateau in PD accumulation, a consistently high PDT (of nearly 37 days), and low expansion rate (<20% per passage) were observed. Mean cell yield per passage in our culture protocol was two-three times lower for ADSC (Table 2).

 The growth curves (Figure 1(d)) depict the kinetics of proliferation within a single passage. Both cell types follow a typical sigmoid growth pattern but with different phase durations. WJSC exhibit a lag phase similar to that of ADSC (about 2.5 days) but a much shorter log phase (3.5 as opposed to 7.5 days) during which they expand more promptly (about fivefold expansion of cell population). WJSC become confluent by day 7, while they continue to grow by 15%-20% even when superconflluent, forming dense cultures of compacted cells, whereas ADSC undergo contact inhibition after day 11 not exceeding 90% confluency. GM containing the dipeptide form of glutamine increases cell yield during log phase by up to 200% (especially for ADSC; *P* < 0.01, day 9), while it enhances survival of postconfluent WJSC cultures (*P* < 0.05, day 11; see online Supplementary Figure 2). This is in concordance with the effects of glutamine dipeptide on bone-marrow-derived MSC survival and growth [28].

### 3.3. Phenotypic Characteristics of Long-Term Cultures

**Figure 2:**
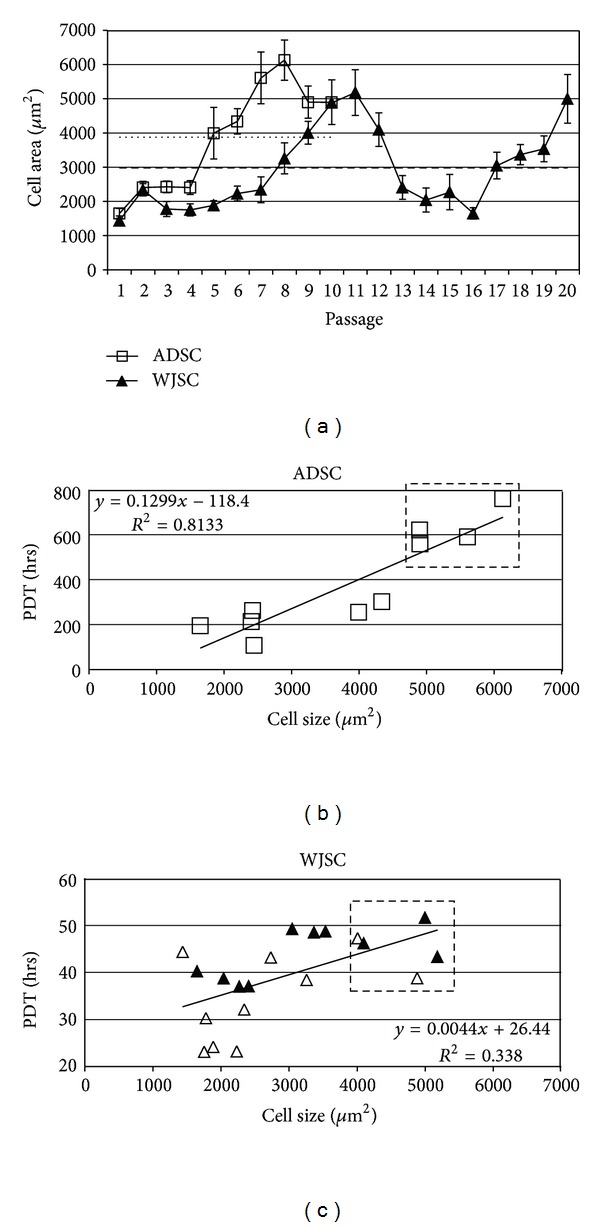
Variation in cell size (surface area in *μ*m^2^) over time in culture and its association with proliferation kinetics. (a) Cell size (mean area ± SEM) as a phenotypic property of WJSC and ADSC during serial propagation. Dashed and long dashed horizontal lines denote mean cell size for ADSC (3876 *μ*m^2^) and WJSC (2926 *μ*m^2^), respectively; (b) regression analysis of ADSC size versus PDT at respective passages (*P* < 0.001, Spearman *r* correlation coefficient = 0.915). Trendline equation and goodness of fit (*R* ^2^) are shown. Boxed data points correspond to ADSC with sizes over 3,880 *μ*m^2^ (top 25% of cells); (c) regression analysis of WJSC size versus PDT at respective passages (*P* < 0.01, *r* = 0.6). Open triangles, p.1–10; solid triangles, p.11–20. Trendline equation and goodness of fit (*R* ^2^) are shown. Boxed data points correspond to WJSC with sizes over 4,600 *μ*m^2^ (top 25%).

**Figure 3:**
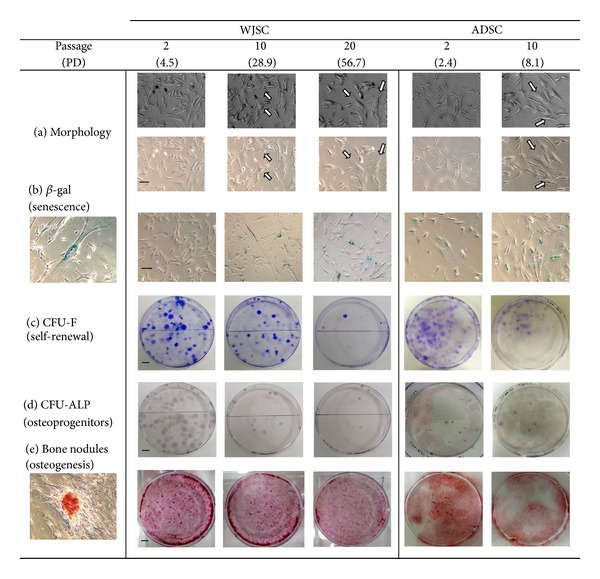
Phenotypic properties of WJSC and ADSC at early and late passages. (a) Light microscopy photos illustrating cell morphology; arrows show cells with type III morphology. Top row, phase contrast. Mag. = 25x. (b) Senescence, as depicted by *β*-gal staining. Mag. = 25x. Subpanel shows a giant cell (>650 *μ*m in length) with typical senescent phenotype. (c) Crystal violet staining of CFU-F (self-renewal capacity). (d) Formation of CFU-ALP (osteoprogenitor colonies). (e) Calcified bone nodule formation in response to osteogenic stimulation. Subpanel illustrates a typical alizarin red-stained bone nodule at high magnification (25x). Scale bars: (a) and (b) 100 *μ*m, (c) and (d) 10 mm, and (e) 4 mm.

Figure 2(a) illustrates the change in cell size, as a phenotypic characteristic, during prolonged subculture. A marked, greather than 300% change in cell size over culture time was observed for both MSC populations. ADSC mean size was 3876 *μ*m^2^, with minimum (1648) and maximum (6130) values observed at passages 1 and 8, respectively (Figure 2(a)). WJSC had an average size of 2926 *μ*m^2^ (min 1437, max 5183, at passages 1 and 11, resp.; Figure 2(a)). WJSC exhibited a periodicity in cell size that might be attributed to small, rapidly proliferating cells undergoing rounds of symmetric and asymmetric divisions, which on one hand give rise to differentiating, daughter cells that gradually increase in size and probably become senescent (see also Figures 2(c) and 3), while at the same time replenishing the culture with more subsets of small rapidly proliferating stem cells. In support of this is the association of kinetics (PDT) with cell size (Figures 2(b) and 2(c)). Spearman analysis showed that small cell size correlates better with low PDT, while cells that take longer to undergo rounds of division (senescent) tend to be larger. Specifically, the cells with sizes in the top 25% (arbitrarily denoted as giant cells) have also the highest PDT and the latter correspond to mid to late passages (p.7 or greater for ADSC and p.9 or greater for WJSC).

 Besides cell size, a marked change was observed in other phenotypic properties of MSCs over *in vitro* expansion.  Figure 3 outlines these differences between early cultures and culture endpoints. In terms of morphological characteristics, three main morphologies were observed: (a) spindle-shaped, elongated (type I); (b) polygonal, with few, small, membrane protrusions (type II); (c) flattened, with irregular shape and large (frequently multiple) nuclei (type III). Early-passage (p.2) WJSC and ADSC have mainly type II and type I morphologies, respectively (see Table 3 and Figure 3(a)). Serial passaging of both MSC populations results in gradual acquisition of type III morphology, which especially in the case of WJSC increases by 2.8-fold in occurrence and becomes predominant by passage 20. In these culture points, cells with either type I or type III morphologies accounted for over 70% of the cultures (see Table 3) with at least one-in-three cells being a giant cell (see Methods section for size definition) bearing enlarged, often multiple, nuclei and being largely senescent, staining positive for *β*-gal (Figure 3(b)). The occurrence of senescent cells in ADSC cultures was fairly frequent with roughly one cell out of five staining positive irrespective of the culture point (Table 3). On the contrary, the senescence rate of early WJSC culture which is very low, increases 5-6 fold in later passages, albeit with considerable interculture variability. An interesting observation was also that cytoplasmic granularity of WJSC decreased by 35%–40% with passaging, probably relating to rearrangements in the cytoskeleton and protein synthesis machinery (Table 3 and online Supplementary Figure 3).

**Figure 4:**
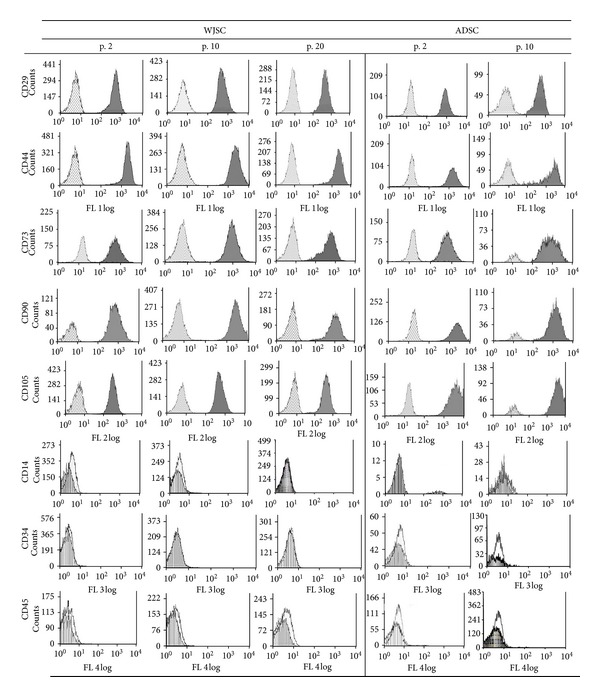
Immunophenotypic analysis of WJSC and ADSC for the expression of typical mesenchymal and hemopoietic stem cell surface markers (first five and last three markers, resp.) during subculture. The vertical axes of the histograms depict event counts, and the horizontal axes represent fluorescence intensities of surface-bound conjugates, that is, marker expression levels. Staining of WJSC and ADSC with specific monoclonal antibody conjugates is illustrated by solid dark histograms (MSC markers: CD-29 and -44 FITC; CD-73, -90, and -105 PE) and by open histograms (HSC markers: CD-14 and -34 ECD; CD-45 PC5). Stripped histograms correspond to isotype-matched (negative) controls.

 In terms of stemness properties, both cell types have a capacity for clonogenicity/self-renewal and lineage-specific differentiation and express a typical immunophenotypic profile, with high MSC marker (>97% positive, except CD44 of late-passage ADSC which was slightly lower at 92 ± 2.3%) and low HSC marker expression (<3% positive, with the exception of 8 ± 0.2% of early-passage ADSC which expressed monocyte-related CD14) (Figure 4). The most avidly expressed markers (high signal intensity) were CD44 (H-CAM) and CD105 (endoglin), for WJSC and ADSC, respectively. But although expression levels of CD surface markers remained constant over time in culture, clonogenicity was reduced. For WJSC, about 1 in 45 cells was clonogenic, and this ability diminished by less than twofold over the first 10 passages (+24 PD), but almost by a factor of five in the next 10 passages (+28 PD) (Figure 3(c) and Table 3). ADSC formed robustly clones at all densities tested, however the of CFU-F ratios were lower than those of WJSC that had undergone many more PD. Differentiation capacity/induced lineage specific differentiation (osteoblasts) also diminished with ageing in culture (Figures 3(c) and 3(d), Table 3). For WJSC, again, this decline was more acute for the last 10 passages, although spontaneous differentiation to CFU-ALP increased as a percentage relative to CFU-F, probably a sign of relative lineage commitment/restriction. In passage-2 cultures, calcium concentration was marginally higher for ADSC, probably owing to the presence of bigger and better mineralized nodules, but was undetected in later passages.

 Last, it is worth noting an increased resistance to trypsin digestion for ADSC. The time period required for 50% detachment at passage 4 was 10 min (60%) longer for ADSC as compared to WJSC; passaging did not significantly increase trypsinization time (see online Supplementary Figure 4). Since the cells have similar sizes (at respective passages), it is suggested that the increased resistance to trypsinization may be due to differential adhesion molecule expression.

## 4. Discussion 

MSC populations share common characteristics; however phenotypic diversification is bound to exist, especially in an *in vitro* environment. Indeed, microarray studies show that phenotypic diversification can actually in many cases be attributed more to differential adaptation to *in vitro* culture conditions than to inherent donor sample heterogeneity due to age, sex, pathology, and so forth [29]. In the present study we have focused on the *in vitro* characterization of postnatal human MSC populations originating from sources that differ both developmentally, as well as anatomically, that is, cells isolated from the matrix (Wharton's jelly) of the fetal UC tissue (WJSC) [14] and from the abdominal adipose tissue of adults (ADSC) [15]. We have chosen these two diverse populations, not only because they are representative of the adult and fetal human stem cell phenotype, allowing comparisons to be made, but also for practical reasons, since—in contrast to other MSC populations—high numbers can be relatively easily isolated from the respective tissues with no (as in the case of WJSC) or minimal donor site morbidity. We then serially propagated these two MSC types over an extended period, under standardized culture conditions, in an effort to investigate whether and to what extent they sustain their proliferative capacity and phenotypic stability. 

With regard to ADSC, their isolation is uncomplicated and efficient especially for smaller tissue samples, providing on average about two million cells per gram lipoaspirate. Establishment of primary cultures was, however, not 100% efficient and propagation was slow. Culture threshold was pinpointed at passage 5, up to which the cells divide on average every eight days giving a total of 5.2 PD in a 40-day period. This equates, following serial passaging, to 1.03 × 10^8^ cells enough for a single administration to an 80 kg patient. This yield is comparable to that reported by other studies focusing on ADSC [27], as well as to the yield obtained by the expansion of other adult MSC populations (e.g., BM-MSCs) [30]. Naturally, as in the case of other adult MSCs, such as peripheral blood- and BM-MSCs, ADSC can be used autologously following *ex vivo* expansion. Moreover, they bear the additional advantage of probably being the most abundantly available and readily obtainable MSCs, compared to the aforementioned cell types. A comparative characterization of ADSC and various fetal and adult stem cell populations has been accounted by a number of studies; however a comprehensive comparison to WJSC, especially with respect to long-term culture, is lacking. 

As highlighted earlier, an accumulating number of publications show that fetal (prenatal, as well as perinatal) stem cells bear advantages, in comparison to their adult counterparts. These concern increased proliferation rates, prolonged capacity for differentiation, enhanced sensitivity to chemical stimuli in the extracellular milieu, and adaptation to culture conditions [8, 31–33]. Moreover, there is strong evidence that fetal tissue MSCs are hypoimmunogenic and can be well tolerated in allogeneic transplantation [1, 34]. Possible sources of fetal MSCs that can be easily harvested perinatally are the embryologically derived placenta, umbilical cord (UC) tissue, and UC blood. Historically, MSCs from cord blood have been the most frequently studied and compared with fetal MSC types mainly due to their popularity in stem cell banking [6, 7]. However, their erratic isolation efficiency and cumbersome culture render them unattractive for clinical applications [7, 35]. In contrast, in recent years, Wharton's jelly (WJ), the mesenchyme-like cushioning material found between the vessels of the UC, has been highlighted as one of the compartments of the UC tissue that harbors a particularly large population of MSCs (here termed WJSC) [14]. These cells can be easily grown in culture without the need for specific growth factors or feeder layers, even in xeno-free culture conditions [36, 37], while at the same time they have been found to secrete soluble factors that are supportive of hemopoiesis and ESC growth [38–41]. 

Results from our study clearly suggest that WJSC possess superior proliferation capacity and grow more predictably than adult ADSC. In particular, they have a steeper log phase and a short PDT (a mean PDT of nearly 32 hrs in the first 10 passages is substantially shorter even when compared to cancer cells and hESC with average PDT of 46 and 72 hrs, resp. [42, 43]). It is worth highlighting that most publications describe experiments with MSCs propagated up to passage five. Our data indicate that the theoretical total yield after five passages (provided that all cells are serially subcultured) is 120 times higher for WJSC. Furthermore, with a PDT of just over 24 hrs in these early passages, they divide seven times faster than ADSC. With respect to WJSC, the seventh passage was identified as a culture threshold. By this stage the cells have accumulated 22 PD in 33 days which equate to a total yield of 1.65 × 10^12^ cells, sufficient for 4,000–20,000 *in vivo* administrations. These kinetics are significantly faster than an earlier study on WJSC which reported similar cumulative PD over a three-month period [44]. With respect to the culture period required for getting a minimum number of WJSC for transplantation, this was half than in the case of ADSC. All the above render WJSC preferable for laboratory experimentation as well as for clinical application. With respect to MSCs-based gene therapy, for example, WJSC have been found to incorporate and express transgenes with high efficiency and increased yield, allowing for a better clinical outcome than their adult counterparts [34, 45].

In terms of phenotypic traits, both populations exhibited stable expression of typical MSC surface markers over time. Nevertheless they also showed a gradual loss of self-renewal, differentiation capacity, and acquisition of signs of senescence (gradual change of morphology to type III, increase in size with higher occurrence of giant, and *β* gal positive cells). This suggests that the expression *pes se* of typical CD markers used for MSC characterization is not conclusive for defining “true” stemness and should be used in tandem with other assays. This has also been highlighted in the case of adult BM-MSCs [46]. For ADSC the main phenotypic changes related to increase in size, partial change in morphology, and a decline in clonogenicity. For WJSC, the changes were more dramatic in the late culture points, which, nevertheless, correspond to many more rounds of division in roughly the same culture period compared to ADSC. The characteristics of phenotypic drift (changes in morphology, cytoplasmic complexity, and resistance to trypsinization) are probably associated with underlying changes in cellular metabolism (e.g., depletion of ATP levels), expression of cell adhesion molecules, and rearrangement of the cytoskeleton [10, 47]. Clearly, any in-depth discussion regarding mechanisms based on our data is largely speculative and beyond the scope of this paper. More work is required in that direction and, for example, whole-genome transcriptomics to detect global changes in gene expression, followed by verification of selected markers (associated with cell cycle, stemness, and lineage-specific differentiation) by real-time PCR, will aid in the dissection of the molecular mechanisms mediating phenotypic drift. Additionally, evaluation of telomere shortening, expression of hTERT, and karyotypic analysis for mutations might reveal critical points in culture with respect to cellular ageing. Combined, these analyses may even provide important information on whether the drift is reversible, for example, by modification of the culture conditions. 

 In conclusion, WJSC, especially in the first six-seven passages, not only exhibit obvious quantitative advantages but also seem to possess superior qualitative traits (clonal ability, etc.) compared to ADSC. Owing to their high subcultivation efficiency and ease of manipulation in culture WJSC feature as ideal candidates for use in cytotherapy protocols. Before their ultimate use in the clinic though, further optimization of *ex vivo* expansion is required, for example, by maintaining cultures in chemically defined media free of animal products and by employing the use of clinical-grade bioreactors or suitable three-dimensional scaffolds [48, 49]. Furthermore, better validation of their therapeutic potential is needed by carefully designed *in vivo* studies using suitable animal models. With respect to this our group is currently working towards establishing protocols that minimize heterogeneity in MSC-based cancer cytotherapy studies using mouse models. On the contrary, the clinical applicability of ADSC seems questionable, especially in those cases where considerable scaleup of the ADSC-based tissue-engineered product would be required; this has also been highlighted in a recent review [50]. Nevertheless it is possible that there is a potential for boosting their proliferation capacity and prolonging their stem properties by modifying standard culture conditions, for example, by using a hypoxic environment or alternative media supplements (sera, etc.) [51–53]. In any case, establishing a standardized culture environment and dissecting the molecular mechanisms associated with retention of MSC phenotypic identity are pivotal not only for harnessing the full potential of these cells *in vitro*, but also for a better understanding of key differences in their biology.

## Supplementary Material

The supplementary online material includes additional data with respect to efficiency and ease of isolation and in vitro manipulation of the two populations under study. Specifically they relate to estimation of cell yield by measuring physical tissue parameters prior to isolation, enhancement of cell proliferation in the presence of Gln-dipeptide, changes in cytoplasmic properties characteristic of late-passage WJSC, and increased resistance to trypsinization/adherence to substrate for ADSC, irrespective of subculture status.

## Figures and Tables

**Table 1:** Cell isolation and culture establishment efficiency.

	WJSC	ADSC	*P* value
Mean tissue length-weight	13.3 cm ± 2*	173.8 g ± 135.7	—
Mean nucleated cell yield (×10^6^) *(per unit length-weight) *	2.28 ± 1.55 *(0.17/*cm)	312 ± 198 *(1.8/*g)	—
Mean viability at isolation (%)	94.3 ± 2.2	96.6 ± 1.6	>0.05
Mean viability at resuscitation (%)	92.9 ± 4.3	92.7 ± 4.9	>0.05
% of adherent cells in starting culture	17.3 ± 4.6	0.93 ± 0.45	<0.001
Adherent cultures beyond p.3	100% (5/5)	37.5% (3/8)	<0.05
Mean passage-to-passage expansion rate % (p.1–5)	974 ± 317	175 ± 56	<0.01
Mean subculture viability % (p.1–5)	97.6 ± 0.5	96.4 ± 1.9	>0.05

*P* value, WJSC versus ADSC. *A statistically significant association was found between UC tissue sample length and the yield of isolated nucleated cells following tissue processing (see text for details).

**Table 2:** Synopsis of proliferation kinetics data of WJSC and ADSC during long-term *in vitro *expansion.

	Mean PDT (hrs)	Mean PD per passage	Mean cell yield per passage (×10^6^)	Theoretical total yield (×10^8^) after five passages	Average time (days) required for obtaining 80 × 10^6^ cells
WJSC	31.9 ± 6.8 / 38.4 ± 5.2*	2.9 ± 0.63 / 2.8 ± 0.59	3.0 ± 0.82 / 2.25 ± 0.8	120.5	17.8
ADSC	402.3 ± 215.1	0.77 ± 0.24	1.03 ± 0.35	1.03	34.8

WJSC data pairs in the first three columns correspond to cultures in the first 10 passages and in passages 11 to 20, respectively; *P < 0.001. Values in the last two columns have been calculated on the basis of the assumption that all isolated cells are serially subcultivated with no intermediate freezing of cell stocks between successive passages.

**Table 3:** Overview of phenotypic characteristics of ADSC and WJSC at early (p.2) and late (p.10 and p.10/20, resp.) passages.

Passage (cum.PD)	WJSC			ADSC	
	2 (4.5)	10 (28.9)	20 (56.7)	2 (2.4)	10 (8.1)
Mean cell size (*μ*m^2^ ± SEM)	2340 ± 180	4882 ± 646	5003 ± 712	2407 ± 183	4907 ± 653
% giant cells*	0	44 ± 4	30 ± 7	20 ± 5	31 ± 8
Predominant morphology types (%)	II/I (46/38)	I/II (38/32)	III/I (44/36)	I/III (57/26)	I/III (42/33)
Intracellular complexity (SSC)	130	85	77	132	115
Senescence (% *β*-gal positive)	2.1 ± 0.9	11.3 ± 8.3	12.9 ± 11.0	17 ± 6.2	24.6 ± 9.7
Self-renewal (CFU-F: cells seeded)	1 : 46	1 : 73	1 : 366	1 : 68	1 : 140
Osteogenic differentiation					
CFU-ALP: cells seeded *(% of CFU-F) *	1 : 78 *60 ± 6.3 *	1 : 98 *76 ± 2.6 *	1 : 450 *83 ± 1.2 *	1 : 149 *46 ± 6.2 *	1 : 448 *31 ± 1.5 *
Bone nodule formation^ #^	+++	++	+	+++	+
(Ca^2+^) (mg/dL)	1.08 ± 0.04	ND	ND	1.56 ± 0.38	ND

*Giant cells: >3880 *μ*m^2^ (WJSC), >4600 *μ*m^2^ (ADSC).

^#^Bone nodule count scoring: + (<20 nodules), ++ (20–50), and +++ (>50).

ND: not detected.

## References

[1] Chen PM, Yen ML, Liu KJ, Sytwu HK, Yen BL (2011). Immunomodulatory properties of human adult and fetal multipotent mesenchymal stem cells. *Journal of Biomedical Science*.

[2] Nauta AJ, Fibbe WE (2007). Immunomodulatory properties of mesenchymal stromal cells. *Blood*.

[3] Patel SA, Sherman L, Munoz J, Rameshwar P (2008). Immunological properties of mesenchymal stem cells and clinical implications. *Archivum Immunologiae et Therapiae Experimentalis (Warsz)*.

[4] Phinney DG, Prockop DJ (2007). Concise review: mesenchymal stem/multipotent stromal cells: the state of transdifferentiation and modes of tissue repair—current views. *Stem Cells*.

[5] Cavallo C, Cuomo C, Fantini S (2011). Comparison of alternative mesenchymal stem cell sources for cell banking and musculoskeletal advanced therapies. *Journal of Cellular Biochemistry*.

[6] Kern S, Eichler H, Stoeve J, Klüter H, Bieback K (2006). Comparative analysis of mesenchymal stem cells from bone marrow, umbilical cord blood, or adipose tissue. *Stem Cells*.

[7] Montesinos JJ, Flores-Figueroa E, Castillo-Medina S (2009). Human mesenchymal stromal cells from adult and neonatal sources: comparative analysis of their morphology, immunophenotype, differentiation patterns and neural protein expression. *Cytotherapy*.

[8] Zhang X, Hirai M, Cantero S (2011). Isolation and characterization of mesenchymal stem cells from human umbilical cord blood: reevaluation of critical factors for successful isolation and high ability to proliferate and differentiate to chondrocytes as compared to mesenchymal stem cells from bone marrow and adipose tissue. *Journal of Cellular Biochemistry*.

[9] Bongso A, Fong CY, Gauthaman K (2008). Taking stem cells to the clinic: major challenges. *Journal of Cellular Biochemistry*.

[10] Cristofalo VJ, Lorenzini A, Allen RG, Torres C, Tresini M (2004). Replicative senescence: a critical review. *Mechanisms of Ageing and Development*.

[11] Rubin H (1997). Cell aging *in vivo* and *in vitro*. *Mechanisms of Ageing and Development*.

[12] Chamberlain G, Fox J, Ashton B, Middleton J (2007). Concise review: mesenchymal stem cells: their phenotype, differentiation capacity, immunological features, and potential for homing. *Stem Cells*.

[13] Martin I, Baldomero H, Bocelli-Tyndall C, Passweg J, Saris D, Tyndall A (2012). The survey on cellular and engineered tissue therapies in europe in 2010. *Tissue Engineering A*.

[14] Troyer DL, Weiss ML (2008). Concise review: Wharton’s jelly-derived cells are a primitive stromal cell population. *Stem Cells*.

[15] Gimble JM, Katz AJ, Bunnell BA (2007). Adipose-derived stem cells for regenerative medicine. *Circulation Research*.

[16] Puissant B, Barreau C, Bourin P (2005). Immunomodulatory effect of human adipose tissue-derived adult stem cells: comparison with bone marrow mesenchymal stem cells. *British Journal of Haematology*.

[17] Weiss ML, Anderson C, Medicetty S (2008). Immune properties of human umbilical cord Wharton’s jelly-derived cells. *Stem Cells*.

[18] Seshareddy K, Troyer D, Weiss ML (2008). Method to isolate mesenchymal-like cells from Wharton’s jelly of umbilical cord. *Methods in Cell Biology*.

[19] Zuk PA, Zhu M, Mizuno H (2001). Multilineage cells from human adipose tissue: implications for cell-based therapies. *Tissue Engineering*.

[20] Digirolamo CM, Stokes D, Colter D, Phinney DG, Class R, Prockop DJ (1999). Propagation and senescence of human marrow stromal cells in culture: a simple colony-forming assay identifies samples with the greatest potential to propagate and differentiate. *British Journal of Haematology*.

[21] Kaplow LS (1968). Leukocyte alkaline phosphatase cytochemistry: applications and methods. *Annals of the New York Academy of Sciences*.

[22] Beresford JN, Graves SE, Smoothy CA (1993). Formation of mineralized nodules by bone derived cells *in vitro*: a model of bone formation?. *American Journal of Medical Genetics*.

[23] Connerty HV, Briggs AR (1966). Determination of serum calcium by means of orthocresolphthalein complexone.. *American Journal of Clinical Pathology*.

[24] Dimri GP, Lee X, Basile G (1995). A biomarker that identifies senescent human cells in culture and in aging skin *in vivo*. *Proceedings of the National Academy of Sciences of the United States of America*.

[25] Schugar RC, Chirieleison SM, Wescoe KE (2009). High harvest yield, high expansion, and phenotype stability of CD146 mesenchymal stromal cells from whole primitive human umbilical cord tissue. *Journal of Biomedicine and Biotechnology*.

[26] Tsagias N, Koliakos I, Karagiannis V, Eleftheriadou M, Koliakos GG (2011). Isolation of mesenchymal stem cells using the total length of umbilical cord for transplantation purposes. *Transfusion Medicine*.

[27] Zhu Y, Liu T, Song K, Fan X, Ma X, Cui Z (2008). Adipose-derived stem cell: a better stem cell than BMSC. *Cell Biochemistry and Function*.

[28] Sotiropoulou PA, Perez SA, Salagianni M, Baxevanis CN, Papamichail M (2006). Cell culture medium composition and translational adult bone marrow-derived stem cell research. *Stem Cells*.

[29] Wagner W, Wein F, Seckinger A (2005). Comparative characteristics of mesenchymal stem cells from human bone marrow, adipose tissue, and umbilical cord blood. *Experimental Hematology*.

[30] Bruder SP, Jaiswal N, Haynesworth SE (1997). Growth kinetics, self-renewal, and the osteogenic potential of purified human mesenchymal stem cells during extensive subcultivation and following cryopreservation. *Journal of Cellular Biochemistry*.

[31] Christodoulou I, Buttery LDK, Tai G, Hench LL, Polak JM (2006). Characterization of human fetal osteoblasts by microarray analysis following stimulation with 58S bioactive gel-glass ionic dissolution products. *Journal of Biomedical Materials Research B*.

[32] Ramkisoensing AA, Pijnappels DA, Askar SFA (2011). Human embryonic and fetal mesenchymal stem cells differentiate toward three different cardiac lineages in contrast to their adult counterparts. *PLoS ONE*.

[33] Zhang ZY, Teoh SH, Chong MSK (2009). Superior osteogenic capacity for bone tissue engineering of fetal compared with perinatal and adult mesenchymal stem cells. *Stem Cells*.

[34] O'Donoghue K, Fisk NM (2004). Fetal stem cells. *Best Practice and Research Clinical Obstetrics and Gynaecology*.

[35] Manca MF, Zwart I, Beo J (2008). Characterization of mesenchymal stromal cells derived from full-term umbilical cord blood. *Cytotherapy*.

[36] Hartmann I, Hollweck T, Haffner S (2010). Umbilical cord tissue-derived mesenchymal stem cells grow best under GMP-compliant culture conditions and maintain their phenotypic and functional properties. *Journal of Immunological Methods*.

[37] Hatlapatka T, Moretti P, Lavrentieva A (2011). Optimization of culture conditions for the expansion of umbilical cord-derived mesenchymal stem or stromal cell-like cells using xeno-free culture conditions. *Tissue Engineering C*.

[38] Ding DC, Shyu WC, Lin SZ, Liu HW, Chiou SH, Chu TY (2012). Human umbilical cord mesenchymal stem cells support non-tumorigenic expansion of human embryonic stem cells. *Cell Transplantation*.

[39] Fong CY, Gauthaman K, Cheyyatraivendran S, Lin HD, Biswas A, Bongso A (2012). Human umbilical cord Wharton's jelly stem cells and its conditioned medium support hematopoietic stem cell expansion ex vivo. *Journal of Cellular Biochemistry*.

[40] Lu LL, Liu YJ, Yang SG (2006). Isolation and characterization of human umbilical cord mesenchymal stem cells with hematopoiesis-supportive function and other potentials. *Haematologica*.

[41] Magin AS, Körfer NR, Partenheimer H, Lange C, Zander A, Noll T (2009). Primary cells as feeder cells for coculture expansion of human hematopoietic stem cells from umbilical cord blood—a comparative study. *Stem Cells and Development*.

[42] Jeon BG, Kumar BM, Kang EJ (2011). Characterization and comparison of telomere length, telomerase and reverse transcriptase activity and gene expression in human mesenchymal stem cells and cancer cells of various origins. *Cell and Tissue Research*.

[43] Park YB, Kim YY, Oh SK (2008). Alterations of proliferative and differentiation potentials of human embryonic stem cells during long-term culture. *Experimental and Molecular Medicine*.

[44] Jo CH, Kim OS, Park EY (2008). Fetal mesenchymal stem cells derived from human umbilical cord sustain primitive characteristics during extensive expansion. *Cell and Tissue Research*.

[45] Chan J, O’Donoghue K, de la Fuente J (2005). Human fetal mesenchymal stem cells as vehicles for gene delivery. *Stem Cells*.

[46] Rallapalli S, Bishi DK, Verma RS, Cherian KM, Guhathakurta S (2009). A multiplex PCR technique to characterize human bone marrow derived mesenchymal stem cells. *Biotechnology Letters*.

[47] Cho S, Park J, Hwang ES (2011). Kinetics of the cell biological changes occurring in the progression of DNA damage-induced senescence. *Molecules and Cells*.

[48] Cardoso TC, Ferrari HF, Garcia AF (2012). Isolation and characterization of Wharton's jelly-derived multipotent mesenchymal stromal cells obtained from bovine umbilical cord and maintained in a defined serum-free three-dimensional system. *BMC Biotechnology*.

[49] Dennis JE, Esterly K, Awadallah A, Parrish CR, Poynter GM, Goltry KL (2007). Clinical-scale expansion of a mixed population of bone marrow-derived stem and progenitor cells for potential use in bone tissue regeneration. *Stem Cells*.

[50] Locke M, Feisst V, Dunbar PR (2011). Concise review: human adipose-derived stem cells: separating promise from clinical need. *Stem Cells*.

[51] Tunaitis V, Borutinskaite V, Navakauskiene R (2011). Effects of different sera on adipose tissue-derived mesenchymal stromal cells. *Journal of Tissue Engineering and Regenerative Medicine*.

[52] Yang S, Pilgaard L, Chase LG (2012). Defined xenogeneic-free and hypoxic environment provides superior conditions for long-term expansion of human adipose-derived stem cells. *Tissue Engineering C*.

[53] Valorani MG, Montelatici E, Germani A (2012). Pre-culturing human adipose tissue mesenchymal stem cells under hypoxia increases their adipogenic and osteogenic differentiation potentials. *Cell Proliferation*.

