# Supplementary material for: Comparative Evaluation of Human Mesenchymal Stem Cells of Fetal (Wharton's Jelly) and Adult (Adipose Tissue) Origin during Prolonged In Vitro Expansion: Considerations for Cytotherapy
Source: Stem Cells Int. 2013 Mar 3;2013:246134. doi: 10.1155/2013/246134 (PMC3603673; doi:10.1155/2013/246134)
Supplement: Supplementary file 1 — The supplementary online material includes additional data with respect to efficiency and ease of isolation and in vitro manipulation of the two populations under study. Specifically they relate to estimation of cell yield by measuring physical tissue parameters prior to isolation, enhancement of cell proliferation in the presence of Gln-dipeptide, changes in cytoplasmic properties characteristic of late-passage WJSC, and increased resistance to trypsinization/adherence to substrate for ADSC, irrespective of subculture status. [file 246134.f1.docx]

**Online suppl. figure 1**

**a**

**b**
