# Supplementary material for: Comparative Evaluation of Human Mesenchymal Stem Cells of Fetal (Wharton's Jelly) and Adult (Adipose Tissue) Origin during Prolonged In Vitro Expansion: Considerations for Cytotherapy
Source: Stem Cells Int. 2013 Mar 3;2013:246134. doi: 10.1155/2013/246134 (PMC3603673; doi:10.1155/2013/246134)
Supplement: Supplementary file 2 [file 246134.f2.docx]

**Online suppl. figure 2**

**b**

**a**

*

#
