# Supplementary figures and images for: Comparative Evaluation of Human Mesenchymal Stem Cells of Fetal (Wharton's Jelly) and Adult (Adipose Tissue) Origin during Prolonged In Vitro Expansion: Considerations for Cytotherapy
Source: Stem Cells Int. 2013 Mar 3;2013:246134. doi: 10.1155/2013/246134 (PMC3603673; doi:10.1155/2013/246134)

**Online suppl. figure 3**


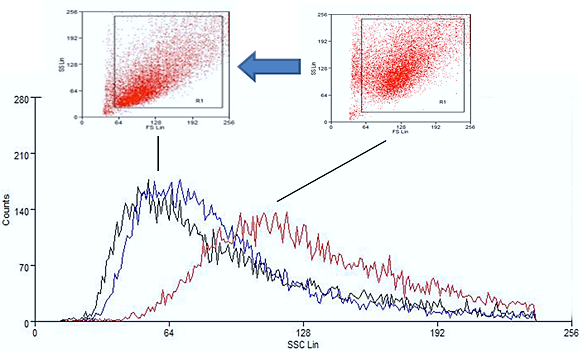


**p.10**

**p.20**

**p.2**

**p.10/20**

**p.2**

Supplement: Supplementary file 3 [file 246134.f3.docx]
